# Pumping up the charge density of a triboelectric nanogenerator by charge-shuttling

**DOI:** 10.1038/s41467-020-17891-1

**Published:** 2020-08-21

**Authors:** Huamei Wang, Liang Xu, Yu Bai, Zhong Lin Wang

**Affiliations:** 1 CAS Center for Excellence in Nanoscience, Beijing Key Laboratory of Micro-Nano Energy and Sensor, Beijing Institute of Nanoenergy and Nanosystems, Chinese Academy of Sciences, Beijing, 100083 China; 2 School of Nanoscience and Technology, University of Chinese Academy of Sciences, Beijing, 100049 China; 3 School of Materials Science and Engineering, Georgia Institute of Technology, Atlanta, GA 30332 USA

**Keywords:** Devices for energy harvesting, Renewable energy, Materials for devices, Nanoscience and technology

## Abstract

As an emerging technology for harvesting mechanical energy, low surface charge density greatly hinders the practical applications of triboelectric nanogenerators (TENGs). Here, a high-performance TENG based on charge shuttling is demonstrated. Unlike conventional TENGs with static charges fully constrained on the dielectric surface, the device works based on the shuttling of charges corralled in conduction domains. Driven by the interaction of two quasi-symmetrical domains, shuttling of two mirror charge carriers can be achieved to double the charge output. Based on the mechanism, an ultrahigh projected charge density of 1.85 mC m^−2^ is obtained in ambient conditions. An integrated device for water wave energy harvesting is also presented, confirming its feasibility for practical applications. The device provides insights into new modes of TENGs using unfixed charges in domains, shedding a new light on high-performance mechanical energy harvesting technology.

## Abstract (web summary)

Conventionally, triboelectric nanogenerators are based on static charges fixed on dielectric surfaces. Here, the authors report a new mechanism using shuttling of mirror charge carriers corralled in quasi-symmetrical conduction domains, which boosts performance for blue energy harvesting.

## Introduction

Entering the era of Internet-of-Things, a revolution on energy supply is desperate to meet the needs of smart, widespread, and wearable electronics^1–5^. Meanwhile, the aggravating damage on our environment caused by fossil fuel motivates the exploration of other types of clean and sustainable power sources to serve as replacement or complement^6–8^. First proposed by Wang in 2012^9^, the triboelectric nanogenerator (TENG, also called Wang generator) presents unique features and great potential as a new technology that converts mechanical energy into electricity, based on the triboelectrification effect and electrostatic induction^10–12^. Driven by the Maxwell’s displacement current, TENGs exhibit superiorities of cost-effectiveness, easy fabrication, high efficiency, lightweight, and versatile material choices^13–18^. In recent years, TENGs have been developing in fields ranging from electronic skin^19^, implantable medical devices^20^, MEMS^21^, plasma instruments^22^, athletic big data^23^ to water quality mapping^24^, wave energy harvesting^25–27^, and so on.

For TENGs, static charges are fully constrained on the dielectric surface, the electric field generated by which induces charge transfer in electrodes when specific motion of the electrodes occurs^12^. A low surface charge density created by triboelectrification greatly reduces their power output and is thereby severely detrimental to their practical applications^14^. Previous studies have focused on material’s selection^28^, structure optimization^29^, surface modification^30^, and environment control^31^ to overcome the problem. Through a common method of corona charging, the charge density of TENGs has been enhanced to 240 μC m^−2^ ^32^. In a high vacuum environment, a charge density of 1003 μC m^−2^ is reached, yet complicated device packaging is needed for practical use^31^. In 2018, a self-charge-pumping TENG adopting the floating layer structure and charge pump was reported, achieving a high effective charge density of 1020 μC m^−2^ even in ambient conditions^33^. However, to fill up the gap towards practical applications, there are still great needs for developing advanced mechanisms in enhancing the output, which should also be facile to be integrated as practical devices working in specific environment.

Here, a high-performance triboelectric nanogenerator based on charge shuttling (referred as CS-TENG) is demonstrated. The CS-TENG introduces a mechanism of charge shuttling in conduction domains. With the interaction of two quasi-symmetrical domains, shuttling of two mirror charge carriers can be achieved to double the charge output. Meanwhile, the charges as the working medium in the domains are effectively generated by a charge pump strategy. An ultrahigh projected charge density of 1.85 mC m^−2^ can be obtained in ambient conditions. Based on the CS-TENG, a high-performance integrated device for wave energy harvesting was fabricated and characterized in water wave environment, successfully demonstrating the technical feasibility of the CS-TENG as a fundamental device to be applied in complex structures for various practical applications. Our work provides insights into new modes of TENGs using unfixed charges in domains, shedding a new light on high-performance mechanical energy harvesting.

## Results

### Structure and working principle of the CS-TENG

**Fig. 1:**
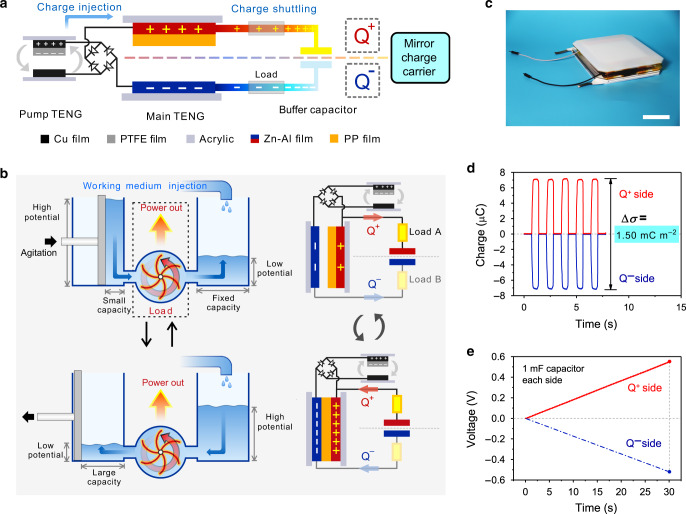
Device structure and working principle. **a** Schematic illustration of charge shuttling. **b** Working process of the charge-shuttling-based triboelectric nanogenerator (CS-TENG) and an analogical model based on hydropower turbine. **c** Photograph of the as-fabricated main triboelectric nanogenerator (TENG) and pump TENG. Scale bar, 4 cm. **d** High output charge density realized in this work restricted by a Zener diode. **e** Demonstration of simultaneously charging two large capacitors. Source data are provided as a Source Data file.

The basic concept of the device is depicted in Fig. 1a. A CS-TENG is mainly composed of a pump TENG, a main TENG and a buffer capacitor. The pump TENG and the main TENG share the structure of a conventional TENG with two electrodes and a dielectric layer^10,12^, whereas the buffer capacitor is a commercialized capacitor. The electrodes of the main TENG and the buffer capacitor form two conduction domains (Supplementary Fig. 1), presenting a quasi-symmetrical structure with Q^+^ side and Q^−^ side. The pump TENG injects charges of opposite signs into these two domains (The extraction of electrons is equivalent to the injection of positive charge carriers) through a rectifier, which should be able to withstand high voltage to block the charges in the domains from flowing back. Upon contact and separation of the main TENG, its capacitance changes while that of the buffer capacitor remains constant, inducing voltage differences between them. Therefore, the charges would be shuttled between the main TENG and the buffer capacitor in a quasi-symmetrical way, generating electricity on the two loads (Supplementary Fig. 1).

The device demonstrates a totally different fundamental mode for harvesting mechanical energy from conventional TENGs, where tribo-generated static charges are fully constrained on the dielectric surface. Here, charge carriers of opposite signs are corralled in two conduction domains (The positive charge carrier is an equivalent of the negative charge moving in the opposite direction). The electric field interaction of corralled charges induces the voltage, which drives charge shuttling in these two domains respectively with a quasi-symmetrical manner. As a result, the charge output ought to be doubled by two mirror charge carriers instead of one. Essentially, the charge shuttling in confining domains represents a peculiar moving pattern of charges. Although here only two domains are presented, they could also be expanded to multiple domains which can be further investigated in the future. Moreover, highly-efficient charge injection by the pump TENG ensures high density of charges in these two domains, up to the limit of dielectric strength. Based on the effect of charge shuttling, it is reasonable to argue that the proposed CS-TENG is highly preferable compared to a conventional TENG whose output is limited by the low density of static charges from triboelectrification. Meanwhile, although the pump TENG is also adopted as a charge source to separate positive and negative charge carriers, the CS-TENG is essentially different from the self-charge-pumping TENG reported previously, which is still based on the fundamental mechanism of conventional TENGs and binds the charges fully in the floating layer like the static charges on the dielectric surface^33^.

To better understand the process of charge shuttling, an analogical model based on hydropower turbine is presented in Fig. 1b. For simplicity, only the situation in one domain is described here, since the other domain works similarly. As shown in the figure, the model presents two connected vessels through a turbine. The left vessel has a variable capacity adjusted by a moving plunger, while the capacity of the right one is constant, corresponding to the main TENG and the buffer capacitor, respectively. A liquid working medium is injected into the vessels, similar to the charge injection by the pump TENG. In the first state of the working process, the rightward movement of the plunger by external agitations reduces the capacity of the left vessel, inducing higher liquid level and potential for the medium inside. The potential can then drive the flow of medium to the right vessel through the turbine, which acts as a load to convert the energy into rotation energy and output power. In the second state, the leftward motion of the plunger will increase the capacity of the left vessel, lowering the potential. Thus, the working medium will flow back via the turbine and output power.

The process of charge shuttling in the CS-TENG is similar to the back-and-forth flow of the working medium. In the device, the injected charges by the pump TENG act as the working medium, whose motion is driven by the electric potential in the domain and generates power output on the load. More specifically, when the main TENG switches to separation state, its capacitance shrinks, and the voltage on it would ascend to a level higher than that on the buffer capacitor. Therefore, charges would flow from the main TENG to the buffer capacitor, passing by the loads and outputting power, until the voltage difference vanishes. When the main TENG changes to contact state, its capacitance grows, causing the voltage on it to descend. Consequently, charges return from the buffer capacitor to the main TENG via the loads.

Figure 1c shows a photograph of the as-fabricated main TENG and pump TENG stacking together. The pump TENG uses polytetrafluoroethylene (PTFE) for the dielectric layer and Cu for the electrode, while the main TENG adopts 5 μm polypropylene (PP) and Zn–Al films. The PP film coated with Zn–Al layer is common for capacitor manufacturing, which is cheap, stable and has high dielectric performance. The purchased buffer capacitor is normally 50 nF unless otherwise specified.

Typical charge output of the CS-TENG in the two domains is shown in Fig. 1d. Simultaneously gathering charges on these two sides gives a high output. A boosted total effective charge density of 1.50 mC m^−2^ is achieved, when a Zener diode is used between the two domains to avoid possible dielectric breakdown. The effective charge density is calculated through dividing the total output of the two sides by the effective contact area, so it is called the projected charge density, which is different from the conventional surface charge density normally referring for TENGs. An ultrahigh projected charge density of 1.85 mC m^−2^ can be obtained without the Zener diode (Supplementary Fig. 2)^33,34^. To further verify the high yield, two large capacitors of 1 mF were simultaneously charged to 0.54 V and 0.52 V, respectively, in 30 s at an agitation frequency of 1.43 Hz (Fig. 1e), suggesting that the output can be utilized to charge or power electronics. Details of the experiment are described in Supplementary Note 1 and Supplementary Figs. 3, 4. The results indicate that the CS-TENG based on charge shuttling can deliver an unprecedented high output for mechanical energy harvesting.

### Electrical characterization of a single CS-TENG

**Fig. 2:**
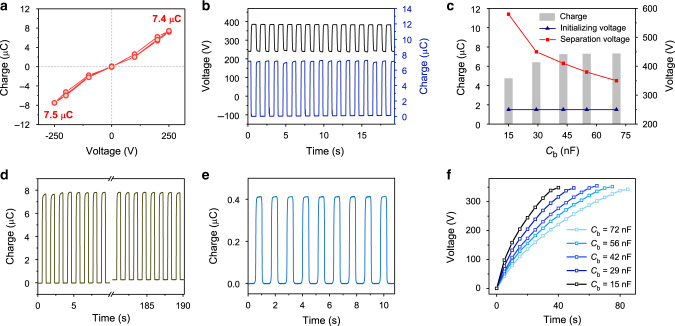
Electrical characteristics of the main TENG and the pump TENG. **a** Shuttled charges of the main TENG in one domain under different initializing voltages. **b** Shuttled charges and voltage of the main TENG with an initializing voltage of 250 V. **c** Shuttled charges and voltages of the main TENG with different buffer capacitors under an initializing voltage of 250 V. **d** Decay of the shuttled charges under an initializing voltage of 250 V. **e** Short-circuit transferred charges of the pump TENG. **f** Voltage curves of the main TENG with different buffer capacitors charged by the pump TENG. Source data are provided as a Source Data file.

To better understand the characteristics of the CS-TENG, the performances of the main TENG and the pump TENG were first characterized individually. For evaluating the output of the main TENG under controlled initial state, a commercial voltage source was used instead of the pump TENG to inject charges, with the main TENG in contact state (Supplementary Fig. 5). The relationship between the initializing voltage on the main TENG and shuttled charges was systematically probed through a cycling test. The initializing voltage started at 0 V and cycled twice from 250 V to −250 V. As expected, the data establish that when the absolute value of the initializing voltage increases, the output rises, reaching a high value of 7.5 μC at −250 V in one domain (Fig. 2a). Little area is enclosed by the curve, indicating that the polarization on the dielectric layer is negligible within such initializing voltage range. Here, only one domain for charge shuttling was measured, since the other domain has similar output. Moreover, although the surface of the PP film can also generate static charges due to contact electrification, they are rather weak compared with the shuttled charges^14^, and are neglected in the analysis.

Details of the shuttled charges and the simultaneous voltage variation by an initializing voltage of 250 V and a buffer capacitor of 50 nF are shown in Fig. 2b. Relevant information on the simultaneous measurement is provided in Supplementary Fig. 6. The voltage of about 240 V in contact state was elevated by separation, driving shuttled charges of 7.2 μC, and reached a steady separation voltage of 385 V. In this process, the voltage of the buffer capacitor would change correspondingly with the pouring-in of charges from the main TENG in separation state. Figure 2c further demonstrates the dependence of the voltage and shuttled charges on the buffer capacitance (*C*_b_) under an initializing voltage of 250 V (Due to that the output with low buffer capacitance is not stable, the data are extracted from the first cycle). As the buffer capacitance decreases from 70 to 15 nF, the separation voltage soars from 350 to 580 V owing to smaller capacitance, and the shuttled charges also shrink, which can be attributed to the high-voltage-induced charge loss in the system. Typically, a voltage higher than about 400 V is likely to implant shuttled charges into the dielectric layer, reducing the charge output. An optimum choice of buffer capacitance should be around 50 nF, considering that larger capacitance would increase the cost and charging time. Besides, the decay of shuttled charges due to charge leakage with time was also checked under an initializing voltage of 250 V. As shown in Fig. 2d, the shuttled charges present a slow decay from 7.8 to 7.53 μC (about 96.5%) in 190 s without continuous charge injection, confirming that a stable and high output of shuttled charges is attainable.

For the pump TENG, the short-circuit transferred charges can achieve 412 nC (Fig. 2e). The relatively large charge output allows fast charging capability. The high peak-to-peak open-circuit voltage of 850 V ensures that the main TENG can be promoted to an adequate voltage (Supplementary Fig. 7). Figure 2f further demonstrates the charging curve of the main TENG with different buffer capacitors using the pump TENG, and the main TENG was kept in contact state in the charging process. It is clear that with the larger buffer capacitance, a slower charging rate is acquired. For a buffer capacitor of 56 nF, it needs about 40 s to charge the main TENG to 250 V on the motor, under an agitation frequency of 0.87 Hz.

**Fig. 3:**
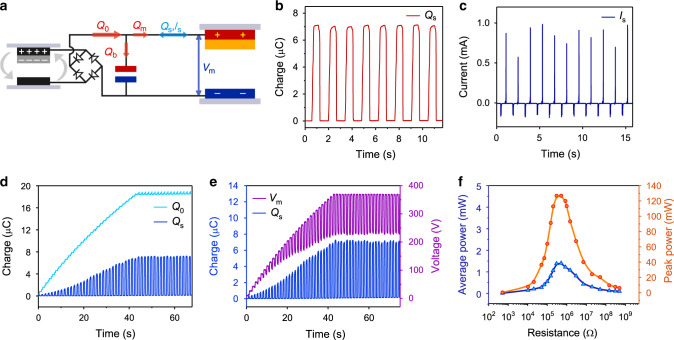
Electrical characteristics of the CS-TENG. **a** Electrical circuit diagram of the experiments. **b**, **c** Shuttled charges (**b**) and corresponding current (**c**) of the CS-TENG. **d** Shuttled charges and simultaneously injected charges of the CS-TENG. **e** Shuttled charges and simultaneous voltage of the CS-TENG. **f** Peak power and average power of the CS-TENG under various loads. Source data are provided as a Source Data file.

Having analyzed the performances of the main TENG and the pump TENG, a CS-TENG was characterized on the motor (Fig. 3a). A Zener diode was applied here for protection. The charge output of the CS-TENG is 7.1 μC in one domain (Fig. 3b), corresponding to a maximum current of over 0.8 mA (Fig. 3c) under an agitation frequency of 0.7 Hz. To further reveal the working mechanism of the CS-TENG, the shuttled charges (*Q*_s_), the total amount of injected charges from the pump TENG (*Q*_0_), and the voltage of the main TENG (*V*_m_) were measured from the very beginning, as shown in Fig. 3d, e. It can be observed that *Q*_s_ and *V*_m_ increase fast from zero with the rise of *Q*_0_. Only a portion of *Q*_0_ is adopted for shuttling, while the rest is deposited in the buffer capacitor. After about 43 s, the voltage reaches a high level approaching 370 V, when the Zener diode takes effect. The voltage no longer rises and no more charges are injected afterwards. At the point, charges of about 18.4 μC are injected, and the maximum shuttled charges are about 7.11 μC. The lower limit of the voltage is about 225 V. A theoretical model on the behavior of the system without the Zener diode is further established, as discussed in details in Supplementary Note 2 and Supplementary Fig. 8. The following equations can briefly describe the output:

1 $$Q_{\mathrm{s}} = \frac{{Q_0}}{{1 + \frac{{C_{\mathrm{b}}d}}{{\varepsilon _{\mathrm{r}}\varepsilon _0S}}}} - \frac{{Q_0}}{{1 + C_{\mathrm{b}}\frac{{d + \varepsilon _{\mathrm{r}}x\left( t \right)}}{{\varepsilon _{\mathrm{r}}\varepsilon _0S}}}}$$

2 $$V_{\mathrm{m}} = \frac{{Q_0}}{{C_{\mathrm{b}} + \frac{{\varepsilon _{\mathrm{r}}\varepsilon _0S}}{{d + \varepsilon _{\mathrm{r}}x(t)}}}}$$

where *d* and *S* are the thickness and area of the dielectric layer (PP), respectively; *x*(*t*) is the separation distance at time *t*; *ε*_r_ is the relative permittivity of PP; *ε*_0_ is the permittivity of vacuum.

The power output of the CS-TENG under different resistances was also tested. As shown in Fig. 3f, a high maximum peak power of 126.8 mW and average power of 1.4 mW are reached at a resistance of 517 kΩ, under an agitation frequency of 1.7 Hz. The power output is highly superior to the conventional TENG, even considering the expense of the pump TENG and the energy consumption of the rectifier^11^. More importantly, the matched load resistance for maximum power output is far lower than that of the conventional TENG^12^, and is more compatible with electronic devices. Thus a much higher powering efficiency can be expected for the CS-TENG in practical applications.

### Structure of the integrated device based on the CS-TENG

**Fig. 4:**
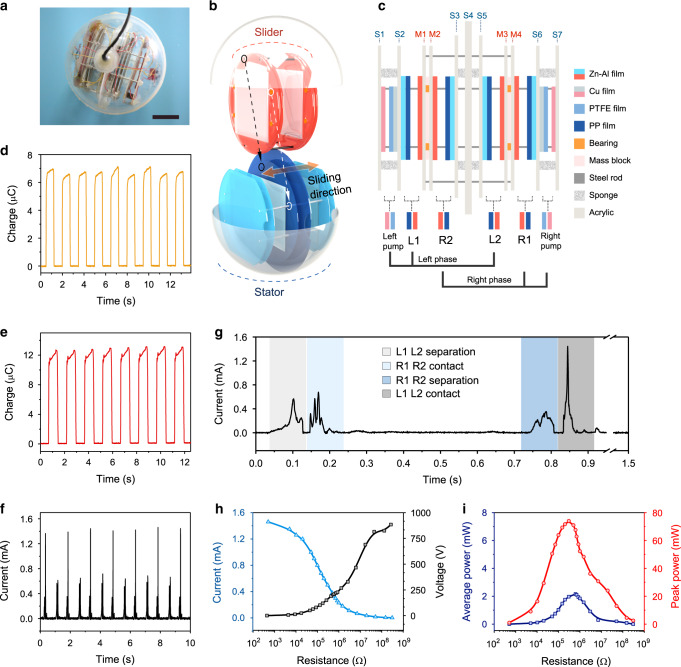
Structure of the integrated device and electrical measurements in air. **a** Photograph of the integrated device based on the CS-TENG. Scale bar, 5 cm. **b** Schematic explosive view of the integrated device. **c** Schematic side view of the integrated device without the package shell. **d**, **e** Shuttled charges of the main TENG L1 (**d**) and main TENGs L1, L2 (**e**) pumped by the left pump TENG. **f** Total output current of four main TENGs in the integrated device. **g** Detailed output current curve extracted from **f**. **h**, **i** Peak current and load voltage (**h**), peak power and average power (**i**) of the integrated device with various loads. The agitation frequency is 0.67 Hz. Source data are provided as a Source Data file.

With the high performance, the CS-TENG is expected to be a fundamental component for developing practical self-powered systems and environment energy harvesters^10,11^. Here, an integrated device for harvesting ocean blue energy of water waves is demonstrated based on the CS-TENG^7,8,25,27,35,36^. As shown in Fig. 4a, b, the integrated device is composed by a slider and a stator encapsulated in a spherical water-proof shell. While the stator is fixed in the shell, the slider can move relative to the stator, in the direction perpendicular to the contact surface, for realizing the contact-separation motion.

More details of the integrated device without the shell are shown in Fig. 4c with a schematic side view. The basic structure of the slider and the stator is mainly a group of acrylic disks (M1–M4 for the slider, S1–S7 for the stator) connected by steel rods. Specifically, disks M1–M4 with mass blocks are fixed with four short rods to form the framework of the slider, and disks S1, S4, S7 are fixed with four long rods as the framework of the stator, on which disks S2, S3, S5, S6 are attached via sponges. Electrodes and dielectric films are adhered on these disks correspondingly, forming four main TENGs (L1, L2, R1, R2) and two pump TENGs (left pump, right pump). With eight bearings, the slider can move smoothly along the long rods of the stator in a reciprocating way, ensuring that each TENG can contact and separate correctly. It is obvious that L1, L2 and the left pump will be in separation state when R1, R2 and the right pump are in contact state, and vice versa. Since the TENGs act in different phases, they are divided into two groups: the left pump driving L1, L2 as the left phase, and the right pump driving R1, R2 as the right phase. Here, the design of one pump TENG driving multiple parallel main TENGs enables a more efficient integration, and the buffer capacitance is also increased from 50 to 100 nF for each phase group with two main TENGs. Moreover, the arrangement of the TENGs also ensures that the main TENGs are in contact state when the corresponding pump TENG acts, facilitating charge injection. Details of the action sequences of the pump TENGs and the main TENGs in the integrated device are described in Supplementary Note 3. The structure guarantees that the pump TENGs and the main TENGs can work consistently even in random agitations based on the relative sliding of the slider.

### Performance of the device for wave energy harvesting

To grasp the fundamental output characteristics under regular agitations, the integrated device was first tested with a linear motor. As shown in Supplementary Fig. 9, the integrated device tilts back and forth periodically as in water waves, causing the slider to move in cycles due to gravity and the inertia force. The charge output of a single main TENG (L1) is more than 6.6 μC (Fig. 4d), while that of a single phase (L1 and L2) has a nearly doubled valuof 12.8 μC (Fig. 4e), at an agitation frequency of 0.67 Hz. The results demonstrate that the integrated device can function effectively under external agitations. Through the rectification shown in Supplementary Fig. 10, the total output of two phases can be collected. Figure 4f, g presents the total rectification current and extracted curve of one period, respectively. It can be observed that there are four peaks in the curve of one cycle, corresponding to the contact and separation actions of the two phases. The nonuniformity can be attributed to the detailed action of each TENG that is indirectly controlled and can affect the current due to $I\;=\;\frac{{dQ}}{{dt}}$^12,37^. The peak current and load voltage under various resistances were also tested, as shown in Fig. 4h. The peak current decreases gradually with the rising resistance, while the voltage increases. The corresponding power output is demonstrated in Fig. 4i. A maximum peak power of 74 mW is achieved at 300 kΩ, and the highest average power reaches 2.16 mW. Compared with that of a single CS-TENG, the decrease of maximum peak power can be ascribed to the changed driving condition for each TENG due to the indirect agitation in the integrated device, where the contact and separation speed of TENGs affects the value of current peaks and corresponding power output^12,37^.

**Fig. 5:**
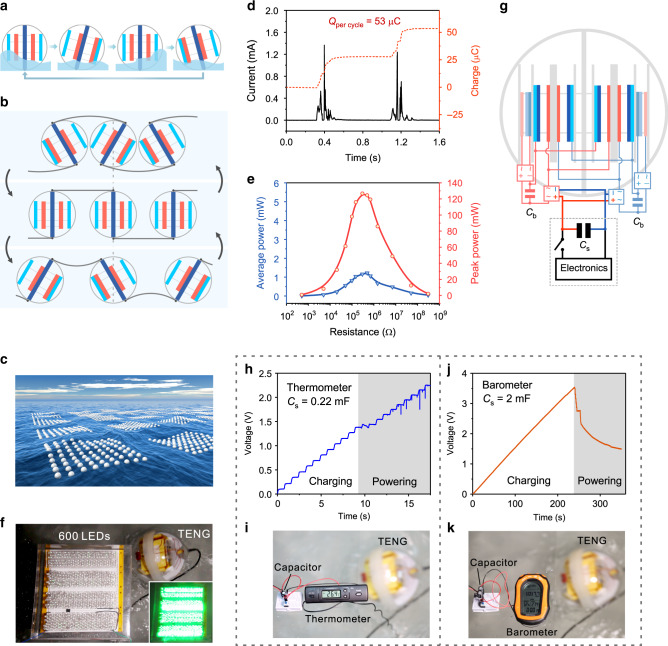
Performance of the integrated device for water wave energy harvesting. **a**, **b** Working principle of a single integrated device (**a**) and networked devices (**b**) in water waves. **c** Schematic perspective of large-scale networks based on the integrated CS-TENG device for harvesting ocean blue energy. **d** Detailed output current and charge curves of the integrated device agitated by water waves. **e** Peak power and average power of the integrated device with various loads in water waves. **f** Directly powering 600 light emitting diodes (LEDs) by a single integrated device. **g** Circuit diagram of the integrated device for powering electronics. **h**–**k** Voltage curves of the storage capacitor and photographs for the integrated device powering a thermometer (**h**, **i**), and a barometer (**j**, **k**). The wave frequency is 0.625 Hz. Source data are provided as a Source Data file.

After preliminary evaluations on the motor, the integrated device was tested in water wave environment of a wave tank. As shown in Supplementary Fig. 11, the integrated device with the water-proof shell can float directly on water surface. The bottom center of the integrated device is constrained by strings tied from two sides of the tank to avoid arbitrary movements. Under agitations of water waves, the device will tilt and restore periodically as shown in Fig. 5a. In real ocean environment, the network structure of integrated devices can be adopted to achieve effective agitations based on the cooperative effect among devices^25^. As presented in Fig. 5b, a zigzag networking strategy using elastic rods to connect neighboring devices can be applied. In such structure, each device has a pair of elastic rods fixed at the top and the bottom, respectively. Under water waves, the design will impose torques on each device via the pair of elastic rods to induce tilt motion, effectively agitating the device. Meanwhile, due to that the output of each integrated device can be rectified first through rectifiers, multiple devices can be easily connected together in parallel for superimposed output, even in random water waves (Supplementary Figs. 12 and 13). Such network can be further augmented for collecting large-scale blue energy with multiple modules, as envisioned in Fig. 5c.

Based on the wave tank experiment, the output performance of the integrated device in water waves was characterized, as shown in Fig. 5d, e. The device can be effectively agitated by water waves and produce a high rectified short-circuit current. The peak current achieves about 1.3 mA with waves of 0.625 Hz, and the corresponding charges by integrating the current reach 53 μC in one cycle (Fig. 5d), which are tens of times larger than previously reported output of typical ball-shell structured devices^25,26,38^. The high charge output is produced via the four main TENGs in the two phase groups and the rectification, which is known to double the charge value by superposing the backward and forward charges in one cycle. The maximum peak power of the integrated device achieves 126.67 mW at 300 kΩ, which corresponds to a volume power density of 30.24 W m^−3^ (The integrated device has a volume of 4.189 × 10^−3^ m^3^), along with a maximum average power of 1.22 mW (Fig. 5e). Actually, based on the design of one pump TENG driving multiple parallel main TENGs, the output could be further enhanced, simply via integrating more main TENGs to each phase^39^, thus even higher output density can be expected. As an intuitive demonstration of the excellent performance, the integrated device is shown to be fully capable of powering 600 light emitting diodes (LEDs) (Fig. 5f and Supplementary Movie 1).

The high output enables the device for constructing practical self-powered systems using water waves as the power source. The corresponding circuit diagram is demonstrated in Fig. 5g. The device first charges a storage capacitor (*C*_s_), which then powers various electronics by closing the switch when the voltage of the capacitor meets the requirement. Figure 5h–k shows as-fabricated self-powered sensing systems for acquiring temperature and air pressure based on the integrated device. For the self-powered temperature sensing, a storage capacitor of 0.22 mF was charged to 1.38 V within 9.1 s (Fig. 5h), then the thermometer without a battery could be driven (Fig. 5i and Supplementary Movie 2). It is notable that after the thermometer is powered, the voltage of the capacitor continues to ascend, demonstrating a redundant energy supply owing to the high output of the integrated device. In the case of self-powered air pressure sensing, two 1 mF capacitors in parallel were charged to 3.54 V in 240 s, before the switch was closed to supply power to the barometer (Fig. 5j). Impressively, the barometer functioned well for longer than 50 s (Fig. 5k and Supplementary Movie 3), confirming the excellent performance of the integrated device based on the CS-TENG. Considering the high output and scalable property, different scales of device networks can be developed and applied for marine self-powered systems and wave energy farms, which are of great value in ocean research and clean energy exploitation.

## Discussion

In this work, the high-performance CS-TENG based on charge shuttling is demonstrated. Unlike conventional TENGs with static charges fully constrained on the dielectric surface, the CS-TENG works based on the shuttling of charges corralled in conduction domains. Driven by the interaction of two quasi-symmetrical domains, shuttling of two mirror charge carriers can be achieved to double the charge output. Moreover, the charges as the working medium can be effectively injected by a pump TENG until the limit of dielectric strength. Based on the mechanism, an ultrahigh projected charge density of 1.85 mC m^−2^ is obtained. With the high-performance CS-TENG, a practical integrated device for harvesting water wave energy is presented, and the design of multiple main TENGs driven by one pump TENG is adopted for efficient integration. Successful operation of the integrated device in water waves with high power output for self-powered sensing confirms the feasibility and flexibility of the CS-TENG in practical applications. The operation frequency of the CS-TENG here is largely restricted by the mechanical aspect of agitating contact-separation motion. In addition to contact-separation mode, the charge-shuttling mechanism can be extended to lateral sliding mode TENGs, and other new modes of TENGs using unfixed charges in domains are also highly expected. As a new fundamental working mechanism, it should be of great value for developing high-performance energy harvesting devices towards practical applications in areas as self-powered systems and blue energy.

## Methods

### Fabrication of the main TENG

Two pieces of acrylic were cut as substrates by a laser cutter (PLS6.75) with dimensions of 120 × 100 × 1 mm. A piece of 100 × 100 × 3 mm silicone rubber (Ecoflex 00-20) was prepared by mixing the base and the curing agent in 1:1 weight ratio and then curing at room temperature for at least 4 h, as a cushion layer attached on one acrylic substrate. A 5-µm thick PP film which was single-side coated with Zn–Al was adhered on the silicone rubber, with the noncoated side to the silicone rubber (The silicone rubber and the PP layer are not shown in Fig. 1a for simplicity). For the other substrate, another Zn–Al coated PP film was assembled on the acrylic with the coated side facing this substrate, producing an effective contact area of 100 × 95 mm.

### Fabrication and integration of the pump TENG

A piece of Cu foil covered by PTFE film (70 mm × 70 mm × 80 µm) was adhered on the opposite side of one substrate of the main TENG. The PTFE film was corona charged with a voltage of 6 kV and a vertical distance of 10 mm between the corona electrode and the PTFE film (ET2673A). Then, a bare Cu foil was adhered on another as-prepared acrylic substrate. Sponges were attached on the substrates to connect the pump TENG, with bonding tapes on the side faces of the substrates to control the separation distance and act as a double protection in case the pump TENG fell apart. The pump TENG and the main TENG were electrically connected through a rectifier which was fabricated using four diodes (typically 1N4007). A Zener diode (3EZ400D5) was used between the positive and negative pins of the rectifier for protection.

### Fabrication of the integrated device

Acrylic disks (2 mm in thickness) and steel rods were fixed by glue to form the framework of the slider and the stator. The rods for the slider are 3 mm in diameter and 71 mm in length, and the rods for the stator are 2.5 mm in diameter and 126 mm in length. TENGs were adhered to corresponding disks. The slider was mounted on the stator with eight bearings (LM3UU) in holes at corresponding disks. The stator was glued to a polystyrene spherical shell (20 cm in diameter), which sealed all the structures.

### Characterization of the device

The shuttled charges, the current and the voltage of the capacitor were measured by an electrometer (Keithley 6514), adding a negligible protective resistance of 500 Ω. A handheld digital oscilloscope (Keysight U1620A) and a multimeter (Victor) were used as supplements for simultaneous measuring. The open-circuit voltage of the pump TENG was measured by an electrostatic voltmeter (Trek 344). A high-voltage DC power supply (DW-P303-1ACF0) was adopted as the voltage source for testing the main TENG without the pump TENG. Eight wave makers (RW-20) were used to generate water waves in a wave tank with a dimension of 1.32 × 0.81 m.

## Supplementary information

Supplementary Information

Description of Additional Supplementary Files

Supplementary Movie 1

Supplementary Movie 2

Supplementary Movie 3

## Data Availability

The data that support the plots within this paper and other findings of this study are available from the corresponding author upon reasonable request. Source data are provided with this paper.

## Source data

Source data
