# Supplementary material for: Pumping up the charge density of a triboelectric nanogenerator by charge-shuttling
Source: Nat Commun. 2020 Aug 21;11:4203. doi: 10.1038/s41467-020-17891-1 (PMC7442790; doi:10.1038/s41467-020-17891-1)
Supplement: Supplementary file 1 — Supplementary Information [file 41467_2020_17891_MOESM1_ESM.pdf]

Supplementary Information for

Pumping up the charge density of a triboelectric nanogenerator by charge-shuttling

Wang *et al.*

Supplementary Figures

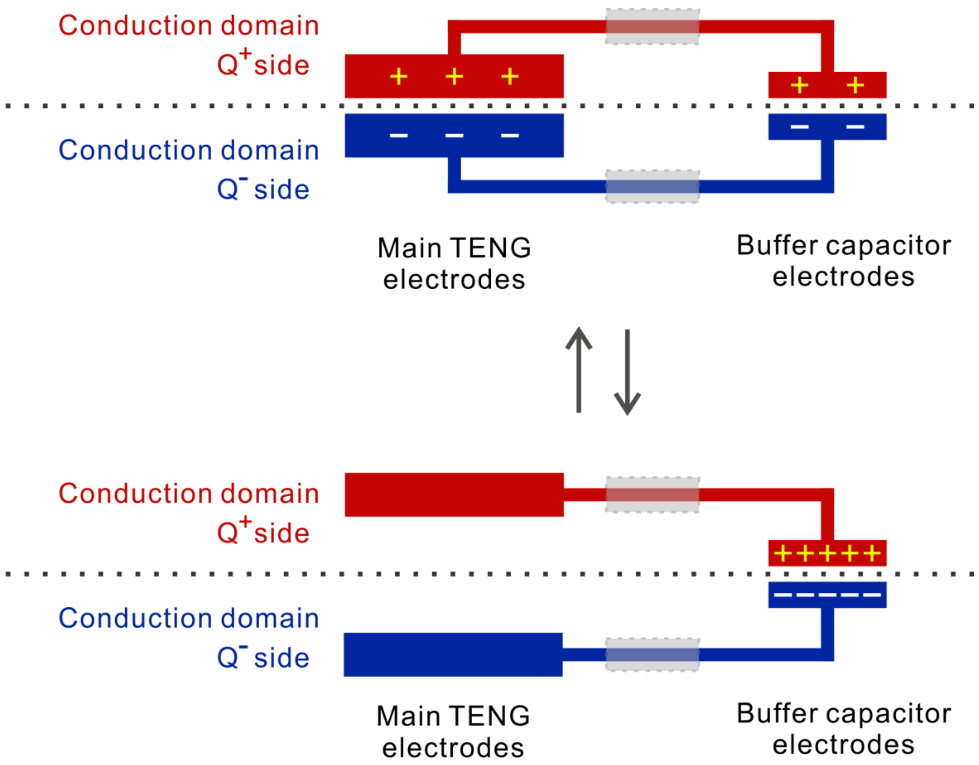

Supplementary Figure 1. Schematic illustration of the two conduction domains.

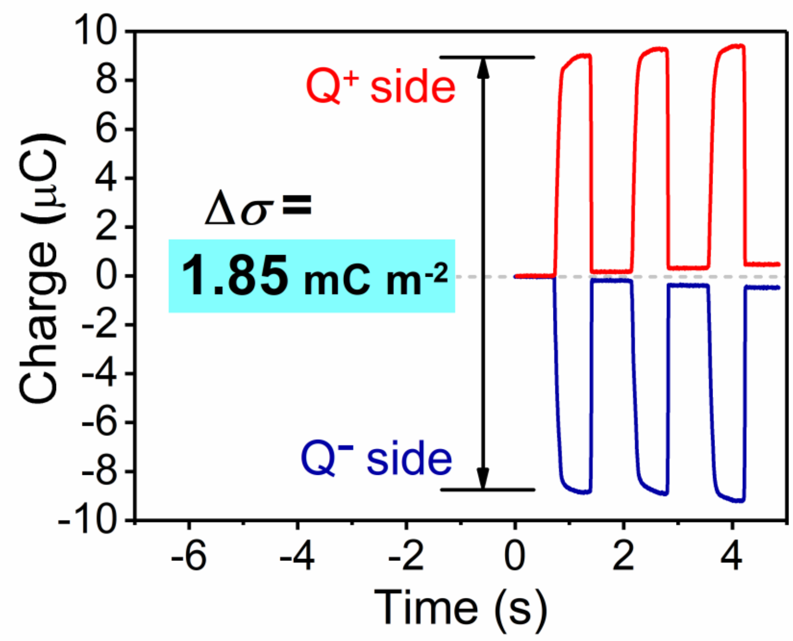

Supplementary Figure 2. Charge density realized in this work without the Zener diode. The charge density is calculated through dividing the total output of the two sides by the effective contact area. The two curves go apart from each other with time due to charge implantation into the dielectric layer, which can be attributed to the high voltage without the Zener diode. Source data are provided as a Source Data file.

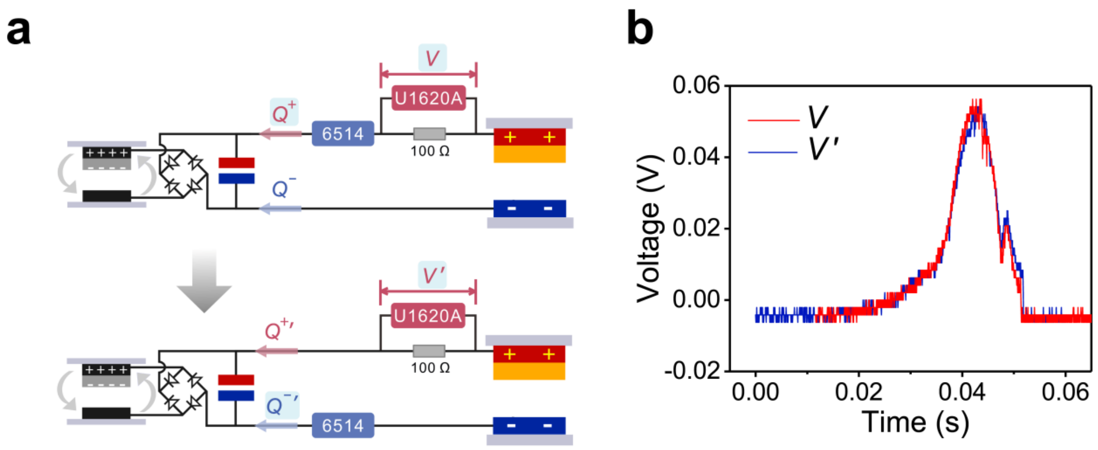

Supplementary Figure 3. Measurement of shuttled charges on both sides. **a** Schematic diagram of steps for measuring the shuttled charges on both sides. **b** Voltage peaks on the probe resistor in the two steps. Source data are provided as a Source Data file.

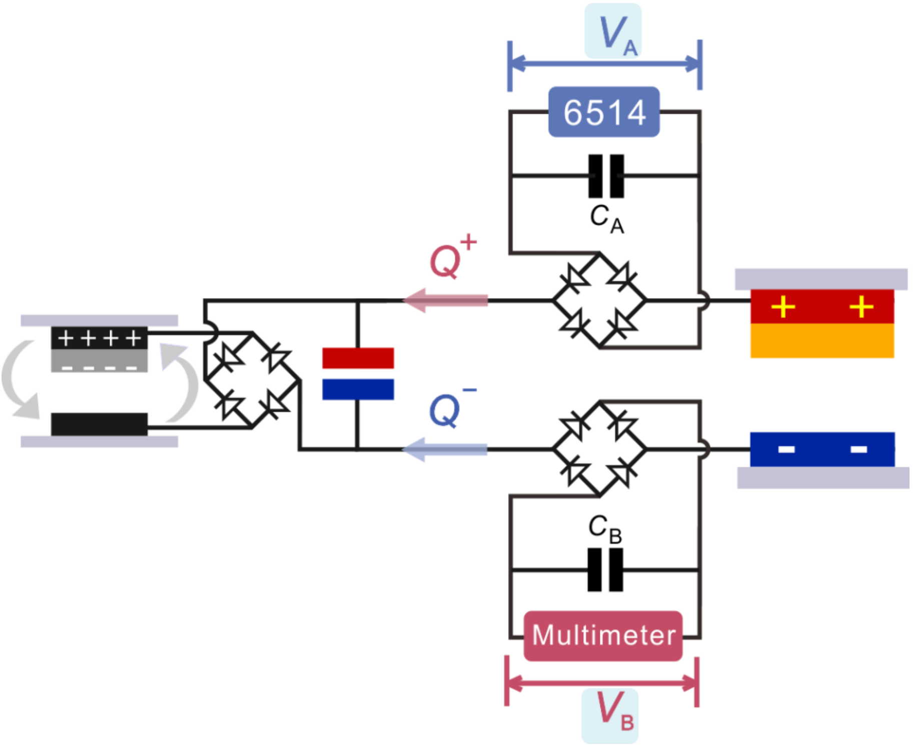

Supplementary Figure 4. Schematic diagram on measuring the voltages of the two capacitors which are simultaneously charged by shuttled charges on the two sides.

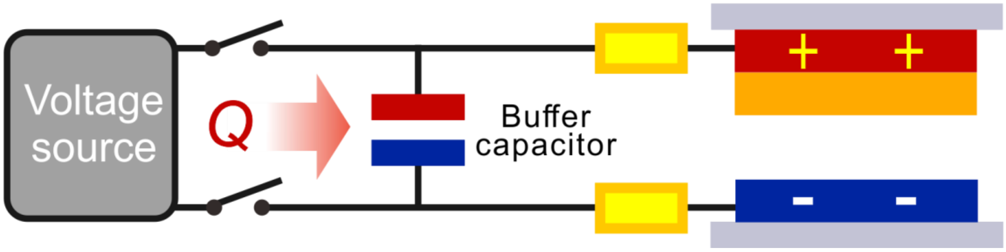

Supplementary Figure 5. Electrical circuit diagram of initializing the device by a voltage source.

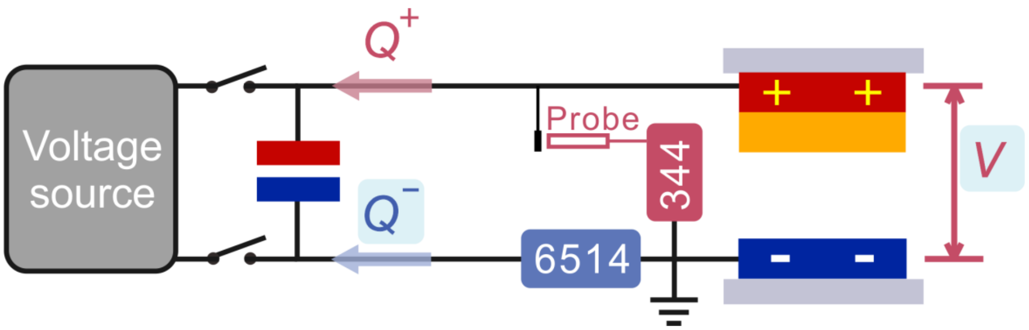

Supplementary Figure 6. Schematic diagram on simultaneously measuring the voltage and shuttled charges of the main TENG, by an electrostatic voltmeter (Trek 344) and an electrometer (Keithley 6514) respectively.

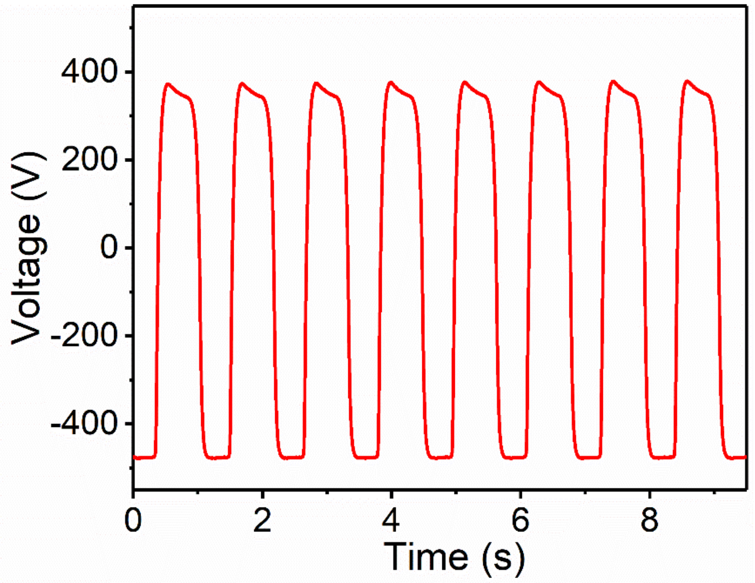

Supplementary Figure 7. Open-circuit voltage of the pump TENG. Source data are provided as a Source Data file.

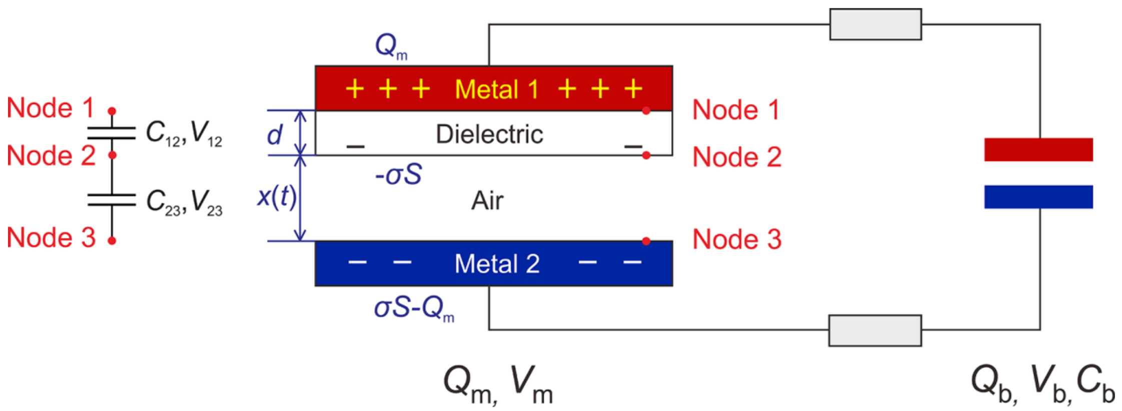

Supplementary Figure 8. Schematic structure and capacitive model of the main TENG.

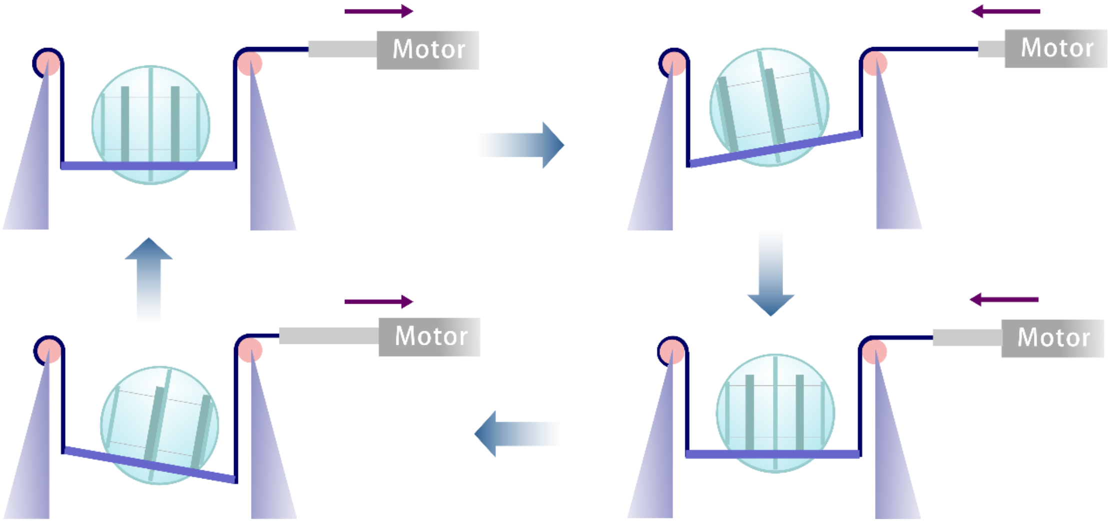

Supplementary Figure 9. Experiment setup of a single integrated device agitated by a motor in air.

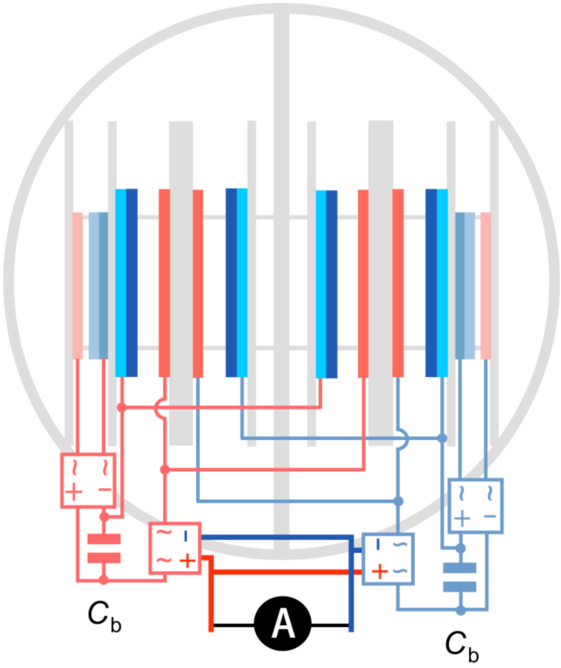

Supplementary Figure 10. Circuit diagram for measuring the total peak current of the integrated device.

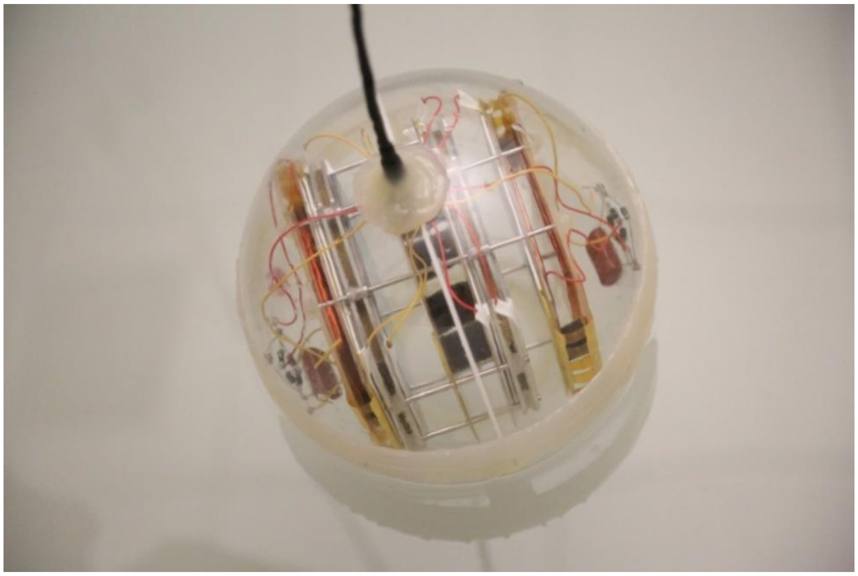

Supplementary Figure 11. Photograph of the integrated device in water.

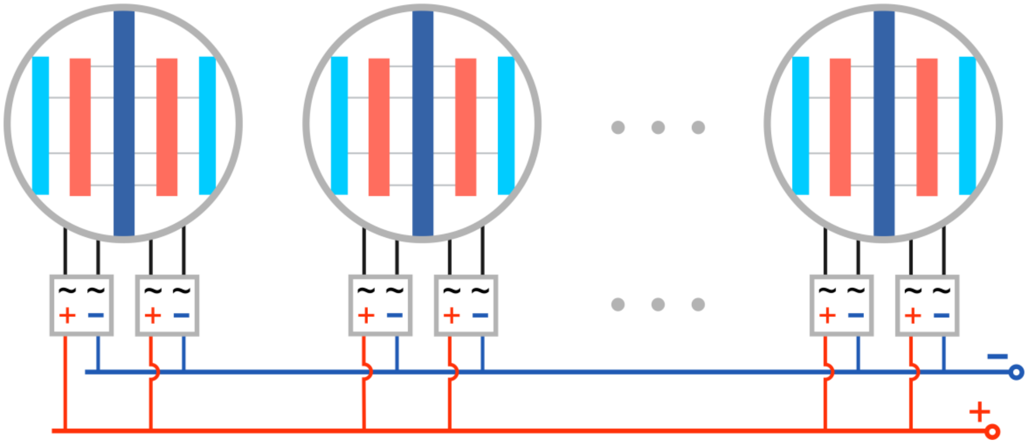

Supplementary Figure 12. Schematic diagram of the rectification circuit for multiple integrated devices.

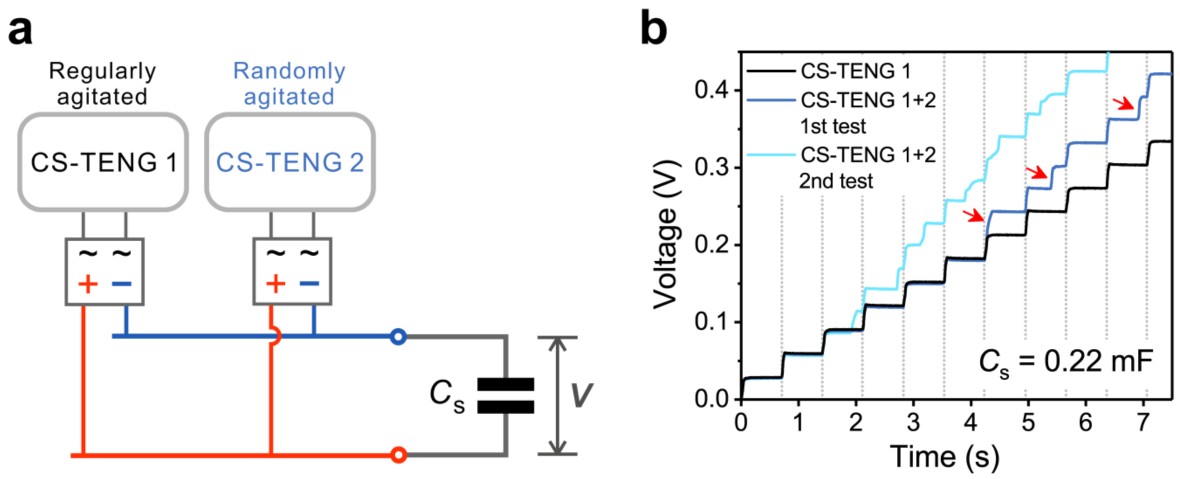

Supplementary Figure 13. Superimposed output of two CS-TENGs agitated independently. **a** Schematic circuit diagram for the rectified output of the two CS-TENGs to charge a capacitor, where CS-TENG 1 is agitated regularly and CS-TENG 2 is agitated randomly. **b** Voltage of the charged capacitor. Due to the large output of the CS-TENG, a step is produced with each contact or separation action of the device. Plot CS-TENG 1 shows a series of regular steps produced by regular agitations. Two plots of CS-TENG 1+2 have extra random steps compared with plot CS-TENG 1, due to the effectively superimposed output of CS-TENG 2 which is agitated randomly. The red arrows indicate the random steps for the 1st test of CS-TENG 1+2. Source data are provided as a Source Data file.

Supplementary Tables

Supplementary Table 1. Parameters for estimating the output.

Parameter	Value
ε_r (PP)	2.2
ε_0	8.854×10^{-12} F m^{-1}
d	5×10^{-6} m
S	9.5×10^{-3} m^2
C_b	5×10^{-8} F
x_{max}	1×10^{-2} m
Q_0	18.4 µC

Supplementary Notes

Supplementary Note 1. Measuring shuttled charges on both sides.

As demonstrated in Supplementary Fig. 3a, the measurement of shuttled charges on both sides was done by two steps with an electrometer Keithley 6514. A digital oscilloscope Keysight U1620A was used to check whether the shuttled charges remained when the electrometer changed its position, by monitoring the voltage on a probe resistor of 100 Ω. The voltage and the charge can be related according to the equation $V = R\frac{\mathrm{d}Q}{\mathrm{d}t}$, where R is resistance and t is time. The floating measurement capability of the oscilloscope U1620A ensures little interference to the circuit, and the small probe resistor of 100 Ω will not affect charge shuttling obviously.

In the first step of the measurement, the electrometer measured the charge output on the Q^+ side, and the oscilloscope monitored the voltage on the probe resistor. After that, the electrometer was shifted to the Q^- side to acquire the charge output of this side, while the oscilloscope kept on monitoring the Q^+ side. As shown in Supplementary Fig. 3b, the voltage peaks in the two steps have almost the same profile, indicating that the charge shuttling does not change obviously with shifting the electrometer. Therefore, the charge output measured by the electrometer in the two steps can be regarded as the shuttled charges on the two sides.

The measurement of the charged capacitors on the two sides is shown in Supplementary Fig. 4. The voltage of the capacitor on the Q^+ side was measured by an electrometer (Keithley 6514), and the voltage of the capacitor on the Q^- side was measured by a multimeter (Victor) which can conduct floating measurement. Both the electrometer and the multimeter were connected parallel to the capacitors. The multimeter was only connected at the end and measured the final voltage to minimize charge leakage.

Supplementary Note 2. Theoretical analysis of the CS-TENG.

Supplementary Fig. 8 shows the schematic structure and capacitive model of the main TENG. The area and thickness of the dielectric layer are S and d respectively. $x(t)$ is the separation distance at time t. By charge injection from the pump TENG, there are charges of Q_m in Metal 1, σS-Q_m in Metal 2, and Q_b in the buffer capacitor that has a capacitance C_b. Residual charges with density σ exist on the lower surface of the dielectric layer due to the charge transfer when the surface contacts with Metal 2, especially in high voltage situations. The amount of residual charges is usually low and can be neglected. The total amount of injected charges is:

$$Q_0 = Q_{\mathrm{m}} + Q_{\mathrm{b}} \tag{1}$$

By defining three equipotential nodes, a capacitive model can be established for the main TENG (Supplementary Fig. 8)[1,2]. Here, Metal 1 and Metal 2 are two equipotential nodes. Another node is the lower surface of the dielectric layer which is also equipotential when assuming that residual charges distribute uniformly. Major capacitances C_{12} and C_{23} exist among these nodes and can be treated as parallel plate capacitors. Due to that the separation distance is normally small, the area can be regarded as large enough to neglect the edge effect. Thus, the capacitances can be expressed as:

$$C_{12} = \frac{\varepsilon_{\mathrm{r}}\varepsilon_0 S}{d} \tag{2}$$

$$C_{23} = \frac{\varepsilon_0 S}{x(t)} \tag{3}$$

where ε_r and ε_0 are relative permittivity of the dielectric film and vacuum permittivity, respectively. The permittivity of air is nearly the same to that of the vacuum. The voltages on the capacitances can be expressed as:

$$V_{12} = \frac{Q_{\mathrm{m}}}{C_{12}} \tag{4}$$

$$V_{23} = \frac{Q_{\mathrm{m}} - \sigma S}{C_{23}} \tag{5}$$

The voltage on the main TENG is:

$$V_{\mathrm{m}} = V_{12} + V_{23} = \frac{Q_{\mathrm{m}}}{C_{12}} + \frac{Q_{\mathrm{m}} - \sigma S}{C_{23}} \tag{6}$$

Based on Supplementary Equations (2) and (3):

$$V_{\mathrm{m}} = \frac{Q_{\mathrm{m}} d + (Q_{\mathrm{m}} - \sigma S)\varepsilon_{\mathrm{r}} x(t)}{\varepsilon_{\mathrm{r}} \varepsilon_0 S} \tag{7}$$

For the buffer capacitor, the voltage is:

$$V_{\mathrm{b}} = \frac{Q_{\mathrm{b}}}{C_{\mathrm{b}}} \tag{8}$$

When there is no load in the circuit, the voltages of the main TENG and the buffer capacitor are equal:

$$V_{\mathrm{b}} = V_{\mathrm{m}} \tag{9}$$

Thus:

$$\frac{Q_{\mathrm{b}}}{C_{\mathrm{b}}} = \frac{Q_{\mathrm{m}}}{C_{12}} + \frac{Q_{\mathrm{m}} - \sigma S}{C_{23}} \tag{10}$$

Considering Supplementary Equation (1):

$$Q_{\mathrm{m}} = \frac{Q_0 + \sigma S \frac{C_{\mathrm{b}}}{C_{23}}}{1 + C_{\mathrm{b}} \left(\frac{1}{C_{12}} + \frac{1}{C_{23}} \right)} \tag{11}$$

Based on Supplementary Equations (2) and (3):

$$Q_{\mathrm{m}} = \frac{Q_0 + \frac{\sigma C_{\mathrm{b}} x(t)}{\varepsilon_0}}{1 + C_{\mathrm{b}} \frac{d + \varepsilon_{\mathrm{r}} x(t)}{\varepsilon_{\mathrm{r}} \varepsilon_0 S}} \tag{12}$$

Due to that σ is usually very small and can be neglected:

$$Q_{\mathrm{m}} = \frac{Q_0}{1 + C_{\mathrm{b}} \frac{d + \varepsilon_{\mathrm{r}} x(t)}{\varepsilon_{\mathrm{r}} \varepsilon_0 S}} \tag{13}$$

The shuttled charges are:

$$Q_{\mathrm{s}} = Q_{\mathrm{m}}(x=0) - Q_{\mathrm{m}}(x) = \frac{Q_0}{1+\dfrac{C_{\mathrm{b}}d}{\varepsilon_{\mathrm{r}}\varepsilon_0 S}} - \frac{Q_0}{1+C_{\mathrm{b}}\dfrac{d+\varepsilon_{\mathrm{r}}x(t)}{\varepsilon_{\mathrm{r}}\varepsilon_0 S}} \tag{14}$$

The maximum shuttled charges are:

$$Q_{\mathrm{s,max}} = \frac{Q_0}{1+\dfrac{C_{\mathrm{b}}d}{\varepsilon_{\mathrm{r}}\varepsilon_0 S}} - \frac{Q_0}{1+C_{\mathrm{b}}\dfrac{d+\varepsilon_{\mathrm{r}}x_{\mathrm{max}}}{\varepsilon_{\mathrm{r}}\varepsilon_0 S}} \tag{15}$$

When the separation distance is large enough:

$$Q_{\mathrm{s,max}} = \frac{Q_0}{1+\dfrac{C_{\mathrm{b}}d}{\varepsilon_{\mathrm{r}}\varepsilon_0 S}} \tag{16}$$

If σ is neglected, with Supplementary Equations (7) and (13), the voltage on the main TENG is:

$$V_{\mathrm{m}} = \frac{Q_{\mathrm{m}}[d+\varepsilon_{\mathrm{r}}x(t)]}{\varepsilon_{\mathrm{r}}\varepsilon_0 S} = \frac{Q_0}{C_{\mathrm{b}}+\dfrac{\varepsilon_{\mathrm{r}}\varepsilon_0 S}{d+\varepsilon_{\mathrm{r}}x(t)}} \tag{17}$$

Thus:

$$V_{\mathrm{m}}(x=0) = \frac{Q_0 d}{C_{\mathrm{b}}d+\varepsilon_{\mathrm{r}}\varepsilon_0 S} \tag{18}$$

$$V_{\mathrm{m}}(x=x_{\mathrm{max}}) = \frac{Q_0}{C_{\mathrm{b}}+\dfrac{\varepsilon_{\mathrm{r}}\varepsilon_0 S}{d+\varepsilon_{\mathrm{r}}x_{\mathrm{max}}}} \tag{19}$$

At the saturation point before the Zener diode takes effect, using parameters from Supplementary Table 1, $Q_{\mathrm{s,max}}$, $V_{\mathrm{m}}(x=0)$ and $V_{\mathrm{m}}(x=x_{\mathrm{max}})$ are calculated as 7.83 μC, 211.5 V and 368 V respectively, coinciding well with the experiment results shown in Figs. 3d and e.

Supplementary Note 3. Details on the working process of the integrated device.

In principle, the pump TENG and the main TENG can be agitated independently. Because the pump TENG only serves as a charge source through the rectifier and has weak coupling with the working process of the main TENG. Once charges are injected into the main TENG by the pump TENG, they are corralled and shuttled in the conduction domains while the main TENG contacts and separates.

However, when the pump TENG and the main TENG work consistently, the efficiency of charge injection can be improved. More specifically, it is better for the main TENG to be in the contact state when the pump TENG acts and injects charges. This is because the injection of charges by the pump TENG through the rectifier is similar as charging a capacitor, and when the main TENG is in contact state, the capacitance will be maximized, thus more charges can be injected under certain voltage. With such consistent working mode, the requirements on the pump TENG can be reduced, which can be optimized using smaller sizes and lower agitation frequencies, and the energy consumption of the pump TENG can also be decreased. The consistent working mode can be easily achieved by simple structural designs as shown in the integrated device in Fig. 4c, which can be agitated similarly like conventional TENGs.

To clarify the consistent working mode, the action sequences of the four main TENGs and two pump TENGs in the integrated device shown in Fig. 4c are described in details here. Firstly, assuming that the slider is at the rightmost potion, the main TENGs R1, R2 and the right pump TENG are in contact state and other TENGs are in separate state. When the device tilts left, the slider will move from right to left relative to the stator. Disks M1-M4 will also move toward left as parts of the slider. The following actions would happen in sequence accompanying the leftward sliding: separation of the right pump when R1 and R2 are still in contact state, by

releasing the sponges behind S3 and S6 → separation of R1 and R2 → contact of L1 and L2 → contact of the left pump when L1 and L2 are in contact state, by compressing the sponges behind S2 and S5. When the device tilts right, the slider will move rightward. Similar actions will happen and complete a full cycle: separation of the left pump when L1 and L2 are still in contact state, by releasing the sponges behind S2 and S5 → separation of L1 and L2 → contact of R1 and R2 → contact of the right pump when R1 and R2 are in contact state, by compressing the sponges behind S3 and S6. It is obvious that each pump TENG only acts when the main TENGs of the corresponding phase are in contact state. The consistent working mode guaranteed by the structural designs ensures that the device can work effectively even in random low-frequency agitations.

Supplementary References

1. Niu, S. et al. Theoretical study of contact-mode triboelectric nanogenerators as an effective power source. *Energy Environ. Sci.* **6,** 3576-3583 (2013).
2. Niu, S. & Wang, Z. L. Theoretical systems of triboelectric nanogenerators. *Nano Energy* **14,** 161-192 (2015).
