# Supplementary material for: Pumping up the charge density of a triboelectric nanogenerator by charge-shuttling
Source: Nat Commun. 2020 Aug 21;11:4203. doi: 10.1038/s41467-020-17891-1 (PMC7442790; doi:10.1038/s41467-020-17891-1)
Supplement: Supplementary file 2 — Description of Additional Supplementary Files [file 41467_2020_17891_MOESM2_ESM.pdf]

Description of Additional Supplementary Files

File Name: Supplementary Movie 1
Description: 600 LEDs are lighted up by the integrated device.

File Name: Supplementary Movie 2
Description: Self-powered temperature sensing based on the integrated device.

File Name: Supplementary Movie 3
Description: Self-powered air pressure sensing based on the integrated device.
